# Inoculation of *Mimosa Pudica* with *Paraburkholderia phymatum* Results in Changes to the Rhizoplane Microbial Community Structure

**DOI:** 10.1264/jsme2.ME20153

**Published:** 2021-03-12

**Authors:** Shashini U Welmillage, Qian Zhang, Virinchipuram S Sreevidya, Michael J Sadowsky, Prasad Gyaneshwar

**Affiliations:** 1 Department of Biological Sciences, University of Wisconsin, Milwaukee, WI 53211, USA; 2 Department of Soil and Water and Climate, University of Minnesota, St. Paul, MN 55108, USA

**Keywords:** Rhizosphere, Nodulation, Inoculation, 16SrDNA

## Abstract

Nitrogen fixing symbiosis between rhizobia and legumes contributes significant amounts of N to agricultural and natural environments. In natural soils, rhizobia compete with indigenous bacterial communities to colonize legume roots, which leads to symbiotic interactions. However, limited information is currently available on the effects of the rhizobial symbiont on the resident microbial community in the legume rhizosphere, rhizoplane, and endosphere, which is partly due to the presence of native nodulating rhizobial strains. In the present study, we used a symbiotic system comprised of *Paraburkholderia phymatum* and *Mimosa pudica* to examine the interaction of an inoculant strain with indigenous soil bacteria. The effects of a symbiont inoculation on the native bacterial community was investigated using high throughput sequencing and an analysis of 16S rRNA gene amplicons. The results obtained revealed that the inoculation induced significant alterations in the microbial community present in the rhizoplane+endosphere of the roots, with 13 different taxa showing significant changes in abundance. No significant changes were observed in the rhizospheric soil. The relative abundance of *P. phymatum* significantly increased in the rhizoplane+endosphere of the root, but significant decreased in the rhizospheric soil. While the rhizosphere, rhizoplane, and root endosphere contained a wide diversity of bacteria, the nodules were predominantly colonized by *P. phymatum*. A network analysis revealed that the operational taxonomic units of *Streptomyces* and *Phycicoccus* were positively associated with *P. phymatum* as potential keystone taxa. Collectively, these results suggest that the success of an inoculated symbiont depends on its ability to colonize the roots in the face of competition by other soil bacteria. A more detailed understanding of the mechanisms by which an inoculated strain colonizes its plant host is crucial for realizing the full potential of microbial inoculants in sustainable agriculture.

Nitrogen-fixing symbiosis between legumes and rhizobia is a major contributor to the global nitrogen cycle (Sprent and James, 2007). We herein use the word rhizobia to collectively refer to all bacteria forming nitrogen-fixing nodules on legumes, regardless of taxonomy. In agricultural systems, these symbiotic relationships have been estimated to contribute more than 80% of fixed nitrogen (O’Hara, 1998). Rhizobial inoculants are extensively utilized to enhance the growth of crop legumes (Deaker *et al.*, 2004; Narożna *et al.*, 2015; Parnell *et al.*, 2016). However, in many cases, these inoculants fail to increase crop productivity due to competition by indigenous rhizobia that are more adapted to the local soil environment (Triplett and Sadowsky, 1992). Rhizobial interactions with host legumes under axenic laboratory conditions have been characterized in detail (Oldroyd *et al.*, 2011; Oldroyd, 2013; Venkateshwaran *et al.*, 2013) and typically start with the colonization of and attachment to plant roots (and sometimes stems) (Poole *et al.*, 2018; Wheatley and Poole, 2018). However, limited information is currently available on the genetic and physiologic traits that are important for inoculated rhizobia that need to survive and compete with other soil microbes to reach and colonize legume roots (Poole *et al.*, 2018). Moreover, soil edaphic factors, the number of indigenous rhizobia, biotic-biotic interactions, and climate all play critical roles in the success of added legume inoculants (Triplett and Sadowsky, 1992). A more detailed understanding of this process is crucial for realizing the full potential of nitrogen-fixing and plant growth-promoting microbial inoculants in commercial agriculture.

The soil immediately in contact with plant roots (the rhizosphere) is rich in microbial numbers and diversity, in large part due to the secretion of substantial amounts of carbon, nitrogenous compounds, and other nutrients into the rhizosphere (Walker *et al.*, 2003; Jones *et al.*, 2009). The majority of the thus far characterized legume-nodulating rhizobia belonging to *Alphaproteobacteria* are metabolically diverse and function well as soil saprophytes in the absence of legumes (Poole *et al.*, 2018). In addition to bulk and rhizosphere soil, other plant compartments and microhabitats impacted by soil bacteria and inoculants include the rhizoplane and endosphere (Clúa *et al.*, 2018; Zhang *et al.*, 2019).

Due to the presence of resident indigenous rhizobia in soil, difficulties are associated with introducing superior inoculant strains (Yates *et al.*, 2011; Parnell *et al.*, 2016; Chibeba *et al.*, 2017) and elucidating the fate of the inoculated rhizobia using the 16S rRNA sequences typically employed in microbial community analyses. Similarly, the majority of other molecular methods cannot differentiate between added inoculant strains and the rhizobia already present in the resident population.

In the last few decades, some strains of *Betaproteobacteria* within the genera *Burkholderia (Paraburkholderia)* and *Cupriavidus* have been identified as symbionts of legumes, such as *Mimosa pudica*. While* M. pudica* and its symbiont (*Paraburkholderia phymatum*) are prevalent in South America and Asia, they have not yet been reported in the Midwestern United States (Gyaneshwar *et al.*, 2011). Soils that lack specific rhizobia provide an opportunity to examine the interactions of an inoculant strain with indigenous bacteria and investigate root colonization *in situ*. A more detailed understanding of these interactions and the mechanisms involved may provide a means to enhance nodulation and subsequently N_2_ fixation by legumes in their non-native environments.

In the present study, we used a soil devoid of indigenous *M.* pudica-nodulating rhizobia to examine host-bacterial interactions in the absence of competition from nodulating strains. We further utilized this symbiosis to assess changes in the bacterial community of *M. pudica* in the rhizosphere, rhizoplane, and endosphere upon an inoculation with *P. phymatum*. The results obtained showed that while the population of *P. phymatum* increased in the rhizoplane of *M. pudica* following the soil inoculation, no significant changes were observed in the bacterial community structure in the soil. Consequently, a plant inoculation with the betaproteobacterium resulted in changes to a limited number of microhabitats.

## Materials and Methods

### Bacterial strains and growth conditions

*P. phymatum* strain MP20 was marked with β-glucuronidase (GUS) as previously described (Gehlot *et al.*, 2013). The strain was maintained on Yeast Extract Mannitol (YEM) agar (Vincent, 1970). In inoculation studies, these strains were grown in YM broth at 28°C with shaking.

### Plant inoculation and growth conditions

The nodulation of *M. pudica* by *P. phymatum* was performed as previously described (Gehlot *et al.*, 2013). Briefly, *M. pudica* seeds (obtained from Outsidepride Seed Source, USA) were treated with concentrated sulfuric acid for 5‍ ‍min, washed five times with sterile water, and germinated on sterile paper towels at 30°C. The soil used in the present study, a Kewaunee mesic Typic Hapludalfs, was obtained from the Cedar Grove Hawk Research Station State Natural Area located in Cedar Grove, WI, (43°33′35.8″N 87°47′55.0″W). To examine plant nodulation in soil, seedlings free of contamination were planted in Styrofoam cups containing a 1:1 ratio of vermiculite and Kewaunee soil. Three seedlings were grown in each cup. On day 2 after transplanting seedlings, three of the cups were inoculated with *P. phymatum* MP20-GUS (~10^6^ bacteria per cup), and another three were used as uninoculated controls. Plants were incubated in a plant growth chamber at 26°C with a 14/10-h light/dark cycle. Plants were watered with modified Jensen’s nitrogen-free medium as needed. In the bacterial community analysis, seedlings were planted in a 1:1 ratio of vermiculite and the same soil, but in plastic trays with drainage. Each tray contained forty seedlings, and three trays were inoculated with independently grown *P. phymatum* MP20-GUS (~10^8^ bacteria per tray). Plants were incubated as described above.

### Plant colonization and nodulation

Plant colonization and nodulation were examined as previously described (Gehlot *et al.*, 2013). Briefly, plants were removed from the soil mix 14 days after inoculation (DAI), and the roots were visually observed for nodule formation. Roots were stained for GUS activity using X-gluc (5-bromo-4-chloro-3-indolyl-β-D-glucuronic acid) to confirm the presence of inoculated *P. phymatum* MP20-GUS. Stained nodules were observed and counted using a dissecting microscope.

### Effects of the inoculant on soil and the rhizosphere microbiome

To examine the effects of the *P. phymatum* inoculation on the bacterial community structure, the rhizosphere and rhizoplane-endosphere soil, roots, and nodules were collected 0, 3, 7, and 14 DAI. Ten plants were taken for each time point (Fig. S1). Briefly, on 0 and 3 DAI, plants were gently removed from the pot, and closely adhering rhizosphere soil was removed with the aid of a sterile spatula. The roots were then separated from rhizosphere soil and homogenized in liquid nitrogen using a mortar and pestle to obtain rhizoplane plus endosphere samples. Regarding samples collected on 7 and 14 DAI, plants were gently pulled from the pot, and the attached rhizosphere soil was collected by gentle shaking and the use of forceps. Tightly adherent soil and the roots were homogenized to obtain rhizoplane samples (this fraction may also contain endospheric microbiota). Nodules were collected on 14 DAI, washed with sterile water, and homogenized. Ground samples were used to isolate DNA with the Qiagen^®^ DNeasy PowerLyzer^®^ PowerSoil^®^ kit (Qiagen) according to manufacturer’s instructions

### 16 rRNA gene amplification and sequencing

In the analysis of microbial communities, the V4 hypervariable region of the 16S rRNA gene was amplified by PCR as previously described (Zhang *et al.*, 2019). PCR conditions were 95°C for 5‍ ‍min followed by 25 cycles at 98°C for 20‍ ‍s, 55°C for 15‍ ‍s, 72°C for 1‍ ‍min, and a final extension at 72°C for 5‍ ‍min. Sequencing was performed using barcoded primers and the dual indexing method at the University of Minnesota Genomics Center (UMGC, Minneapolis, MN, USA) (Gohl *et al.*, 2016). Libraries were sequenced using the Illumina MiSeq platform (Illumina).

### Analysis of bacterial diversity

The 300-nt sequences obtained were analyzed using the QIIME and R programs (McMurdie and Holmes, 2013). Briefly, raw sequence reads were trimmed and processed using SHI7 software (Al-Ghalith *et al.*, 2018). Greengenes ver. 13.8 was used to align the processed sequences using NINJA-OPS (Al-Ghalith *et al.*, 2018). Chloroplast and mitochondrial DNA sequences were removed using QIIME, and filtered data were further rarefied to 15,000 reads per sample for subsequent statistical analyses. Raw sequencing data were deposited in the NCBI Sequence Read Archive (SRA) under Accession Number PRJNA649756.

Microbial diversity values were assessed using alpha-diversity indices (*e.g.*, observed species, Chao1, and Shannon) and were calculated from rarefied sequence data using the *phyloseq* package in R program. Differences in beta diversity were calculated based on Bray-Curtis dissimilarity matrices (Bray and Curtis, 1957). Non-metric multidimensional scaling (nMDS) was used to ordinate samples using *metaMDS*. Comparisons of communities between rhizosphere soils and roots were performed using *ANOSIM*. The influence of inoculated *P. phymatum* on the rhizosphere microbiome was analyzed using the Envift function in the *vegan* package (Oksanen *et al.*, 2020).

### Network analysis to identify potential keystone taxa

The co-occurrence network was generated as described by Zhang *et al.* (2019). Briefly, rare operational taxonomic units (OTUs, those with relative taxonomic abundance values <0.01%) were removed from samples, and the WGCNA package (Langfelder and Horvath, 2008) was used to construct the network using Spearman’s correlation matrix. The random matrix theory (RMT) was used to establish whether 0.791 was the appropriate similarity threshold (Luo *et al.*, 2006). Additionally, to avoid type I errors in the correlation matrix, *P* values were further adjusted using the Benjamini and Hochberg false discovery rate (FDR) of <0.05 (Luo *et al.*, 2006). Network properties were calculated using *igraph* (Csárdi and Nepusz, 2006). Potential keystone taxa were identified as follows: OTUs with degree >5, closeness centrality >0.15, and centrality <0.035 (Berry and Widder, 2014). The network image was generated using *Cytoscape* (Shannon *et al.*, 2003).

## Results and Discussion

### Nodulation of *M. pudica* by *P. phymatum* MP20-GUS in soil

**Fig. 1.**
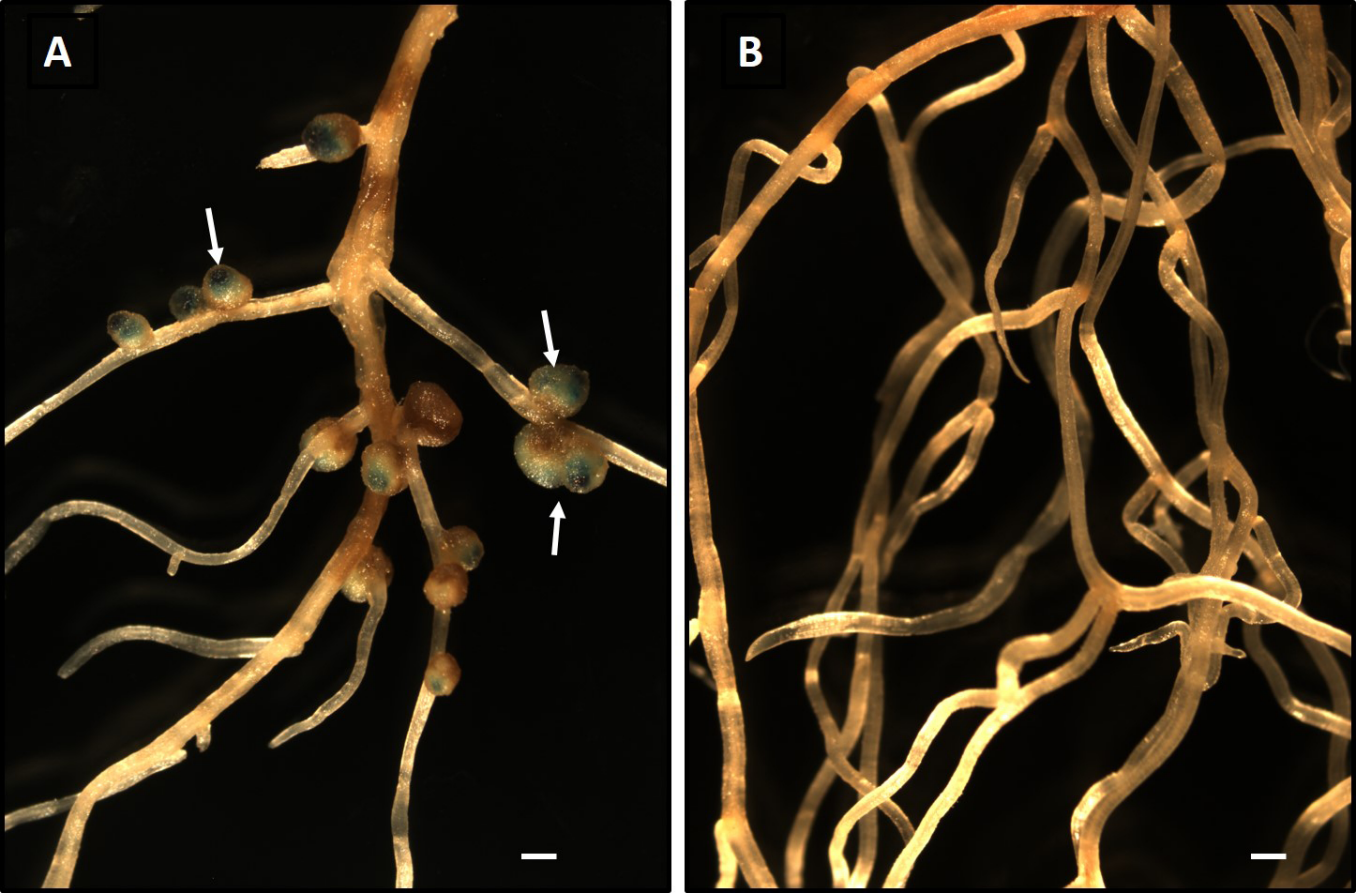
Nodulation of *Mimosa pudica* by *Paraburkholderia phymatum* MP20-GUS in soil. (A) *P. phymatum* formed nodules (arrows) on *M. pudica* roots grown in non-sterile soil. The nodule stained blue due to the GUS activity of the inoculant. (B) No nodules were observed on the roots of non-inoculated plants, indicating a lack of symbionts in the soil. Bar—1‍ ‍mm

To establish whether agricultural soil collected from a field in Cedarburg WI contained rhizobia that nodulate *M. pudica*, farm soil was used as an inoculum for *M. pudica* seedlings, and nodulation was examined after a four-week incubation. No nodules formed on *M. pudica* roots following the soil inoculation (Fig. 1B), indicating the absence of rhizobia that formed a symbiotic relationship with *M. pudica*. These results are consistent with the known native and non-native range of *M. pudica* that does not include the Midwestern USA (Gyaneshwar *et al.*, 2011).

To investigate whether a known symbiont of *M. pudica* competes with microbes in non-native soil and successfully colonizes and nodulates a host legume, we inoculated soil-grown seedlings with approximately 1×10^3^ *P. phymatum* strain MP20-GUS (Gehlot *et al.*, 2013). This bacterium was previously shown to form nodules on *M. pudica* (Gehlot *et al.*, 2013). The inoculation of *M. pudica* with *P. phymatum* MP20-GUS resulted in the formation of nodules throughout the root system (Fig. 1A). To confirm that nodules were formed by the inoculated strain, roots were stained for GUS activity using X-gluc. Blue staining due to GUS was observed in the nodules (Fig. 1A). These results indicated that *P. phymatum* MP20-GUS colonized and formed a symbiotic relationship with *M. pudica* in soil. The colonization of a host plant in non-sterile soils will have to compete with native microorganisms for resources that are present in the plant rhizosphere and recognize signaling molecules in the presence of other microorganisms. The ability of *P. phymatum* to efficiently nodulate *M. pudica* in non-native soil was expected because this bacterium was previously identified as a major symbiont of *M. pudica* in the Indian subcontinent in which *M. pudica* is highly invasive (Gehlot *et al.*, 2013). These findings suggest that *M. pudica*-*P. phymatum* symbiosis in non-native soils may be developed further as a model system to obtain a more detailed understanding of plant-bacterial interactions *in situ* rather than under axenic artificial plant growth conditions.

### Microbial community changes in *M. pudica* rhizosphere and rhizoplane soils following the inoculation.

The soils comprising the plant rhizosphere and rhizoplane are rich in nutrients and, thus, contain a great diversity of microorganisms. Previous studies estimated that 1‍ ‍g of soil contained >10,000–50,000 bacterial species (Roesch *et al.*, 2007; Berendsen *et al.*, 2012; Turner *et al.*, 2013). To establish whether an inoculation with *P. phymatum* leads to specific changes in the resident soil bacterial community surrounding *M. pudica*, we collected rhizosphere and rhizoplane+endosphere soils from the roots of soil-grown *M. pudica* plants with or without the inoculation with *P. phymatum*. The bacterial community structure and dynamics were examined using a 16S rRNA gene sequence analysis.

**Fig. 2.**
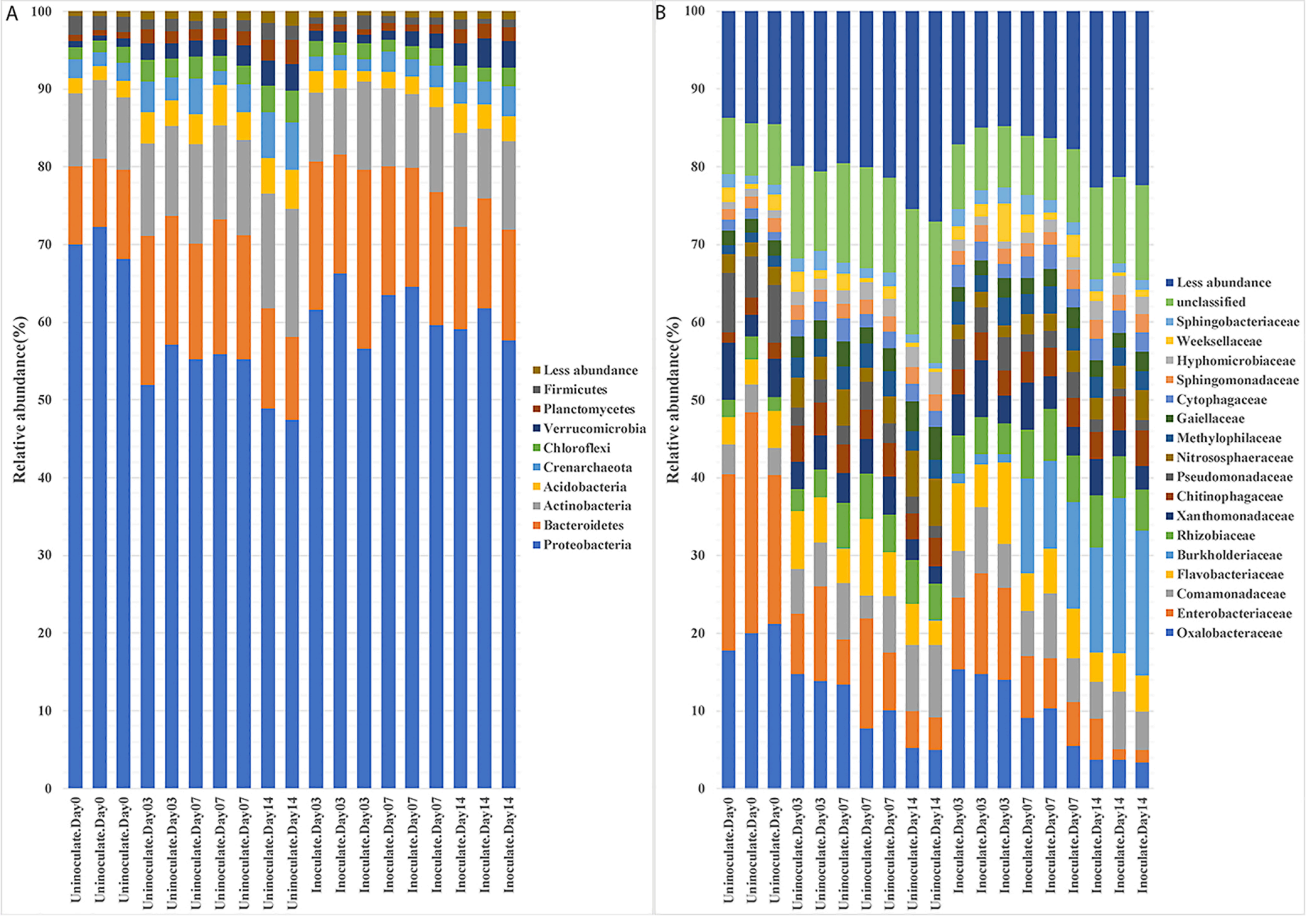
. Relative abundance of OTUs in the rhizoplane+endosphere with or without the *Paraburkholderia phymatum* inoculation on different days after the inoculation. (A) Phylum and (B) family-level classifications. The inoculation led to a significant decrease in *Proteobacteria* and an increase in *Actinobacteria*, *Acidobacteria*, *Crenarchaeota*, *Chloroflexi*, *Verrucomicrobia*, and *Planctomycetes* (*P*<0.05).

In uninoculated plants, the bacterial community associated with the rhizoplane showed changes on different days of growth. While *Proteobacteria* decreased from 70.2 to 48.2% during the two-week incubation, the amounts of *Actinobacteria*, *Acidobacteria*, *Crenarchaeota*, *Chloroflexi*, *Verrucomicrobia*, and *Planctomycetes* significantly increased (Fig. 2A). Previous studies reported that the root microbiome depends on the soil and plant species (Lundberg *et al.*, 2012; Bulgarelli *et al.*, 2013; Tkacz and Poole, 2015). Consequently, the changes observed in the bacterial community during two weeks of growth were consistent with previous findings showing that the plant exerts control over its microbiota via the secretion of specific metabolites from the roots, which change with the growth of the plant (Bais *et al.*, 2006; Lundberg *et al.*, 2012; Bulgarelli *et al.*, 2013).

The inoculation with *P. phymatum* induced a significant shift in the OTUs present in rhizoplane+endosphere samples (Fig. 2B) from those in uninoculated plants. Moreover, OTUs representing 13 different taxa showed significant differences in relative abundance (Fig. 2B). Specifically, OTUs corresponding to *Oxalobacteraceae*, *Enterobacteriaceae*, *Pseudomonadaceae*, and *Sphingobacteriaceae* showed a significant decrease over the two-week period, whereas OTUs representing *Rhizobiaceae*, *Nitrososphaeraceae*, and *Hyphomicrobiaceae* increased (Fig. 2B).

**Fig. 3.**
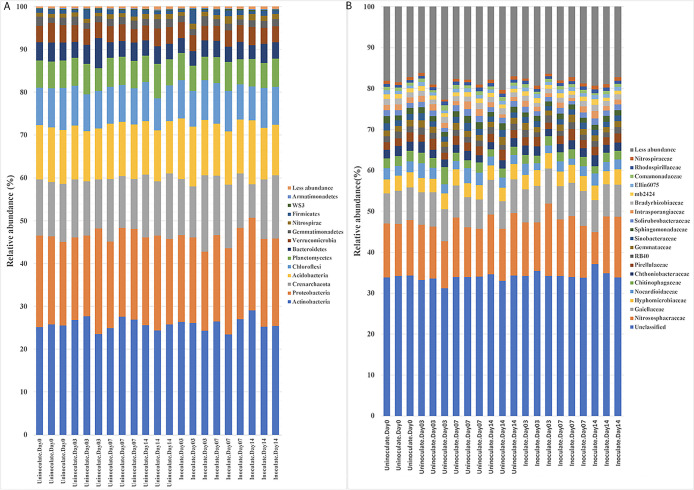
Relative abundance of OTUs in rhizosphere soil with or without the *Paraburkholderia phymatum* inoculation on different days after the inoculation. (A) Phylum and (B) family-level classifications. There were no significant differences (*P*>0.05) between inoculated and uninoculated samples.

In contrast to the changes observed in rhizoplane soil, the inoculation did not induce marked changes in the dynamics of OTUs in the rhizosphere soil (Fig. 3). The most abundant‍ ‍OTUs belonged to *Actinobacteria* (25.88±1.40%), *Proteobacteria* (20.56±1.29%), *Acidobacteria* (12.67±0.81%), and *Crenarchaeota* (13.28±1.92%), (Fig. 3A). Similarly, no major changes due to the *P. phymatum* inoculation were observed when OTUs were characterized at the family level. These diverse OTUs belonged to 19 major families and were dominated by *Nitrosophaeraceae*, *Gaiellaceae*, and *Hyphomicrobiaceae* at 13.3±1.9, 7.9±0.5, and 3.6±0.3%, respectively (Fig. 3B).

**Fig. 4.**
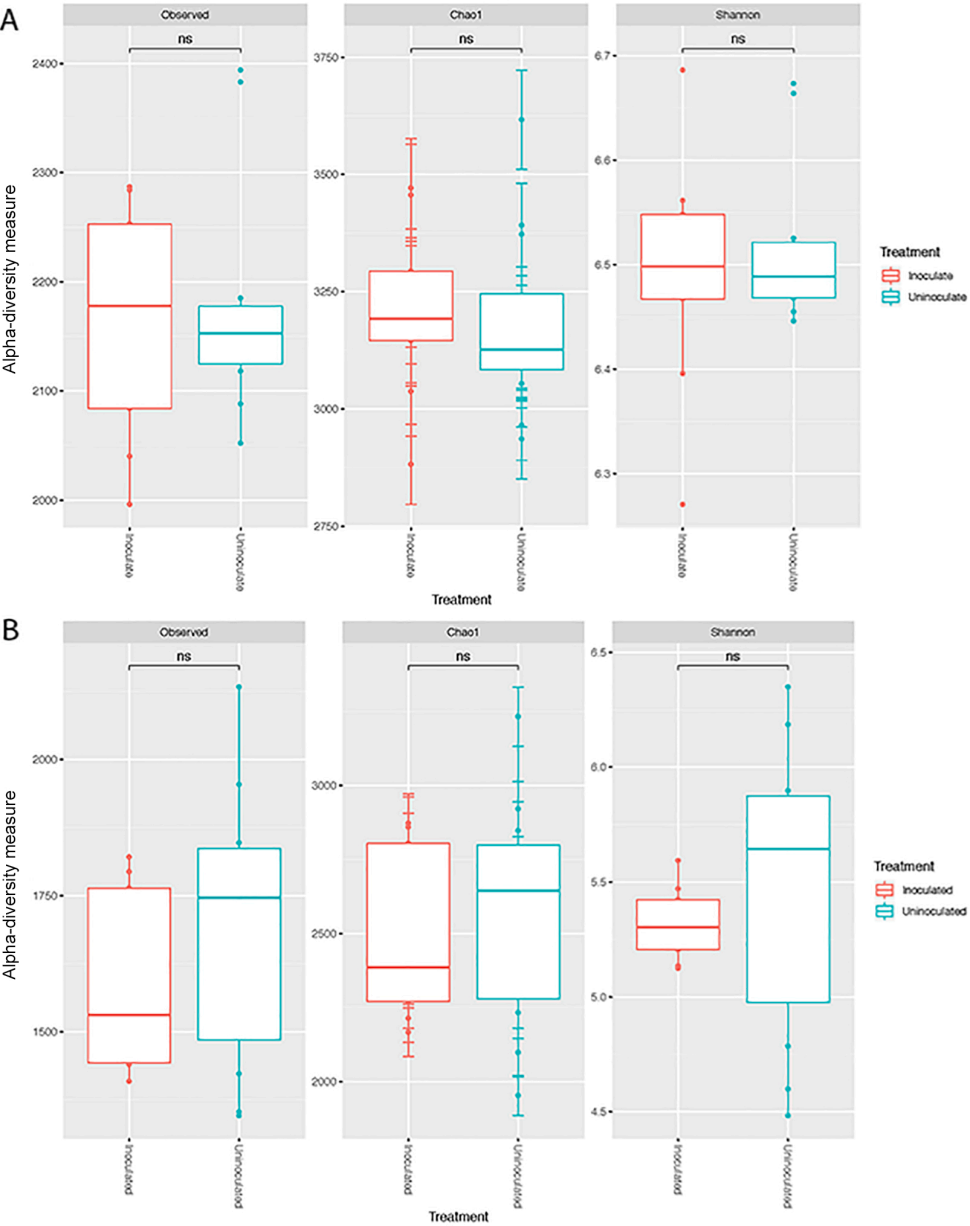
Effects of the inoculation on alpha-diversity. (A) Soil and (B) root. NS—not significant (*P*>0.05).

Although not significantly different (*P*>0.05), alpha-diversity increased in rhizosphere samples, but decreased in the rhizoplane as a result of the inoculation. This is consistent with previous findings showing that microbial communities were less diverse in the rhizoplane/roots than in bulk and rhizosphere soils. This is mainly considered to be due to the release of specific metabolites in root exudates (Berendsen *et al.*, 2012; Lundberg *et al.*, 2012). No significant differences were observed in alpha-diversity between uninoculated and inoculated soils and root samples (Fig. 4A and B).

**Fig. 5.**
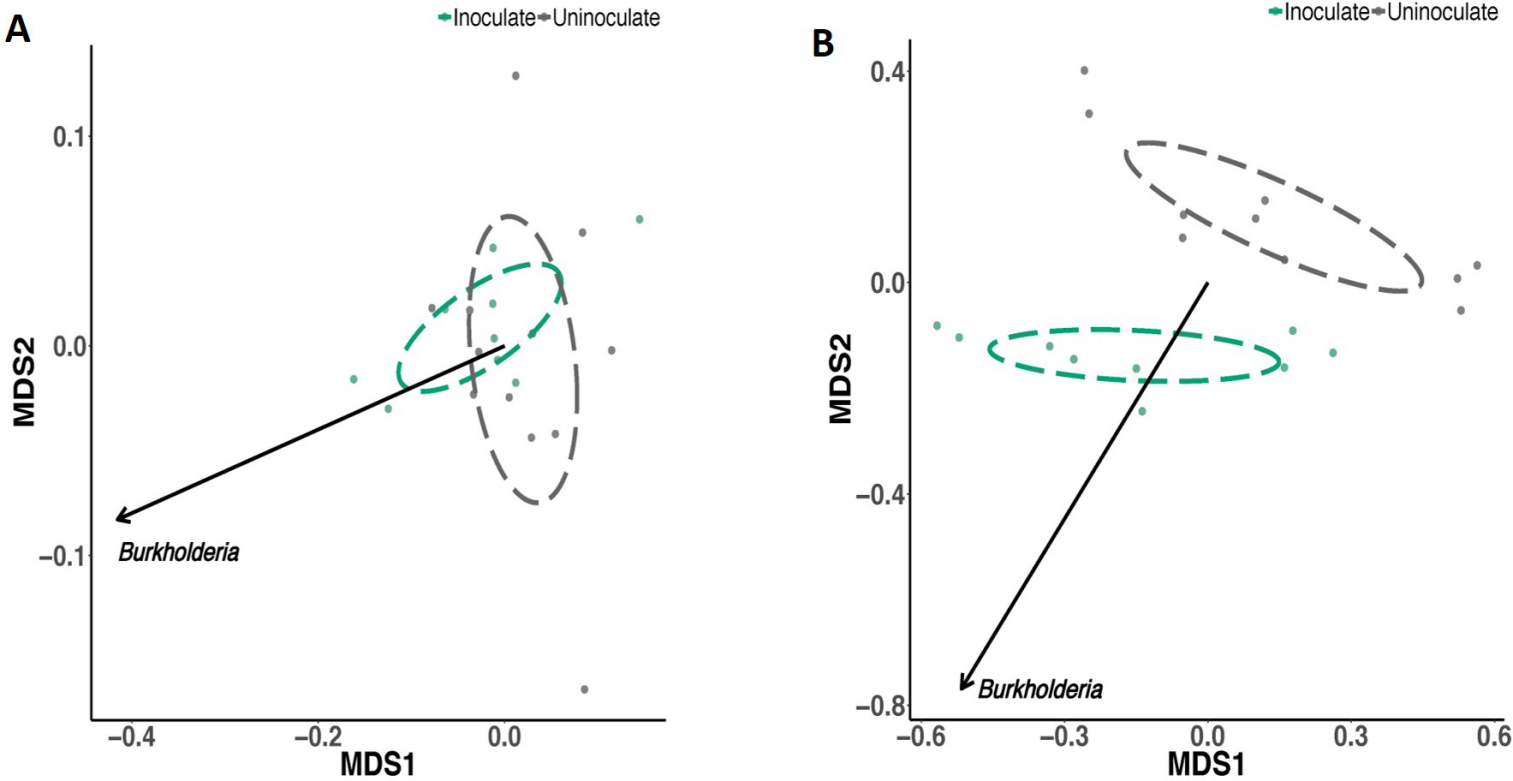
The Bray-Curtis matrix analysis of compositional dissimilarity due to the inoculation. (A) Soil samples showing no significant effects of the inoculation on separation. (B) Root samples showing the clear separation of microbial communities due to the inoculation.

Non-metric multidimensional scaling (NMDS), which represents pairwise dissimilarity between objects, revealed that the inoculation led to the significant separation of communities in rhizoplane+endosphere samples. In contrast, the inoculation did not induce any significant changes in microbial communities in the rhizospheric soil. This was further confirmed using ANOSIM based on an analysis of genera by treatment. The results of the analysis showed that microbial communities were not significantly affected by the *P. phymatum* inoculation (R^2^=0.18, *P*=0.17) in the rhizosphere soil (Fig. 5A), but were significantly separated in rhizoplane/endosphere samples (R^2^=0.86, *P*=0.001) (Fig. 5B).

### Growth of *P. phymatum* in soil

**Fig. 6.**
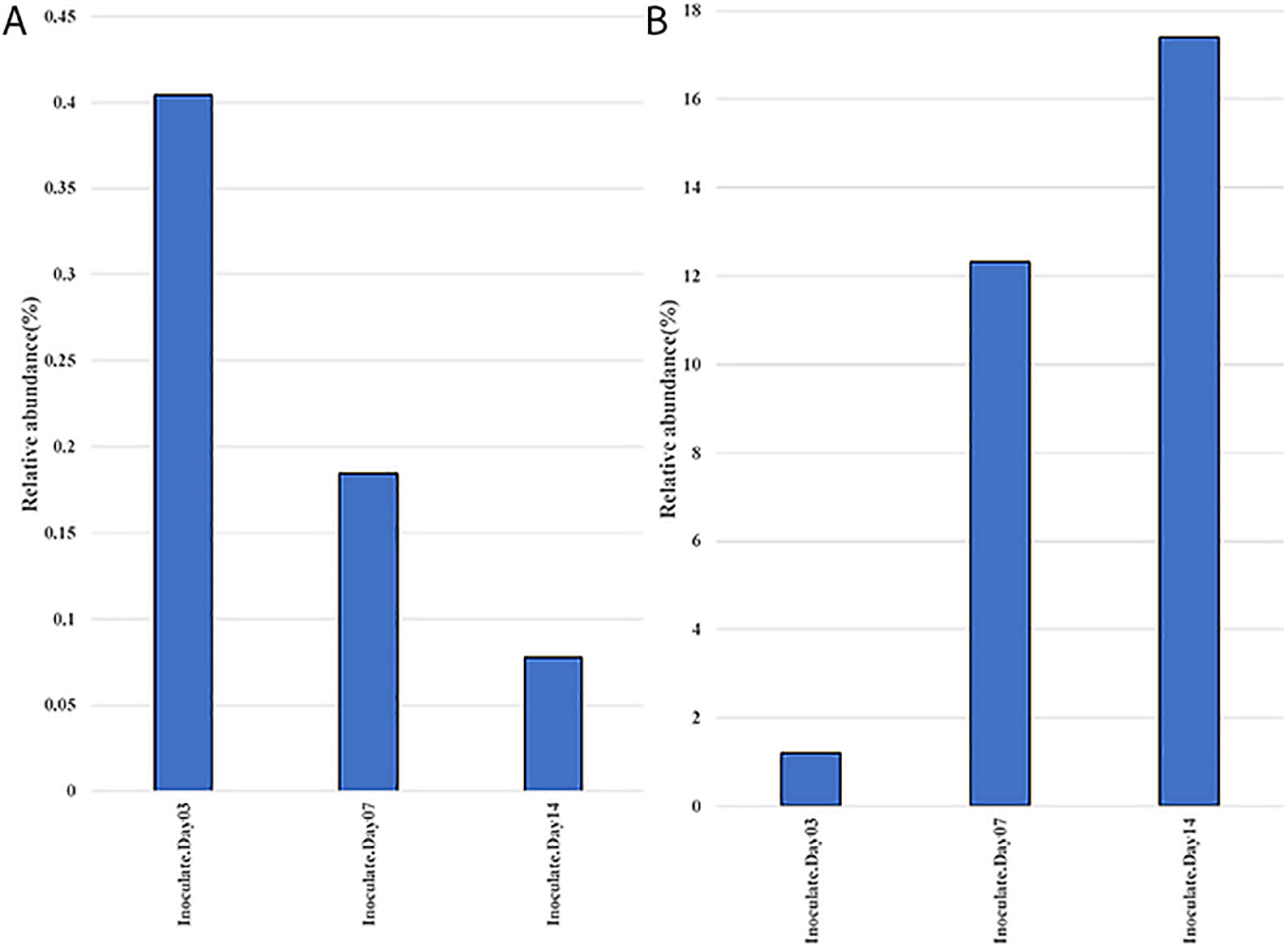
Relative abundance of *Paraburkholderia phymatum* on different days after the inoculation. (A) The *P. phymatum* population significantly decreased (*P*<0.05) in soil, and (B) the *P. phymatum* population significantly increased (*P*<0.05) on/in the root.

The majority of microbial inoculants are generally assumed to multiply in the rhizosphere before colonizing the roots of the plant host. To examine this in more detail, we focused our attention on *Burkholderia*/*Paraburkholderia* OTUs. The results of this analysis (Fig. 6A) showed that *Burkholderia* OTUs significantly decreased over time in the rhizosphere soil from 0.4% at the time of the inoculation to 0.08% by 14 DAI. In contrast, *Burkholderia* OTUs significantly increased in rhizoplane+endosphere samples from 1.2 to 17.4% over the same period (Fig. 6B).

These results suggest that the colonization of the rhizoplane and roots is important for rhizobial competition and growth, more so than just growth in the rhizosphere, which may occur far from the root proper. Root attachment is considered to be most important factor for rhizobial proliferation (Wheatley and Poole, 2018). Root colonization requires the migration of bacteria towards root exudates and the *P. phymatum* genome contains 113 genes annotated as being involved in chemotaxis and motility (Moulin *et al.*, 2014), which were differentially expressed in the presence of *M. pudica* root exudates (Klonowska *et al.*, 2018). *P. phymatum* may utilize *M. pudica* root exudates as nutrient sources more effectively than resident microbes once it colonizes and attaches to the root. A recent study using transcriptomics showed that root exudates of *M. pudica* induced *P. phymatum* genes involved in transport and the utilization of‍ ‍aromatic compounds, inositol, and organic acids (Klonowska *et al.*, 2018). The ability to metabolize myo-inositol was previously shown to be important for root colonization and the nodulation of pea and vetch by *Rhizobium leguminosarum* bv. *viciae* (Fry *et al.*, 2001). Further studies are needed to elucidate the role of these catabolic pathways in the ability of *P. phymatum* to compete with native microorganisms and nodulate *M. pudica* in soil.

### Identification of keystone bacteria affected by the inoculation of *P. phymatum*

**Fig. 7.**
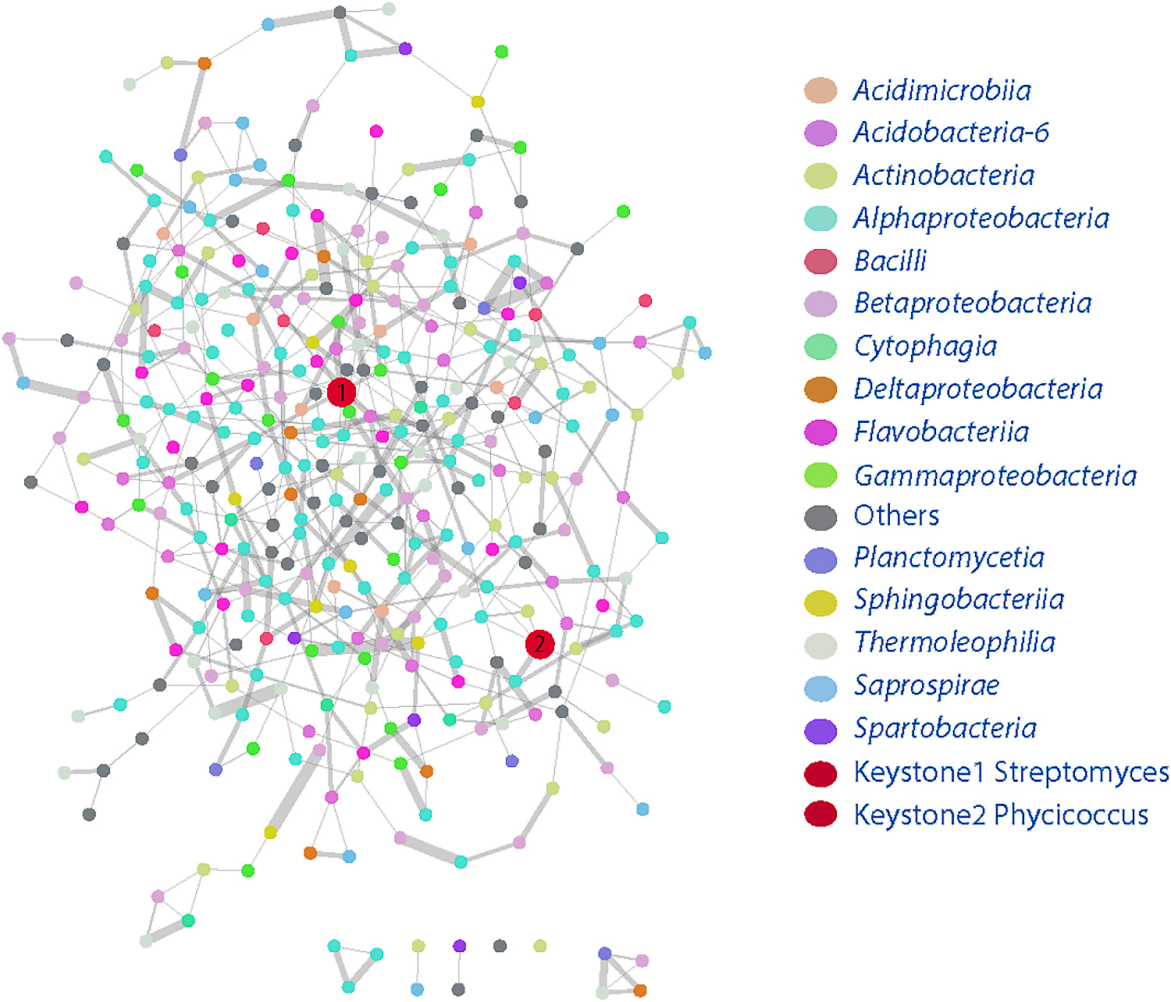
Network analysis of rhizospheric bacterial community associated with the *Paraburkholderia phymatum* inoculation. Nodes represent OTUs, and the size of edges represented the strong (Spearman’s *ρ*>0.791) and significant (*P*<0.05) coorelation between OTUs.

Microbial communities possess different metabolic capabilities and are involved in beneficial and/or competitive interactions with each other. These diverse activities and microbial interactions form functional networks between the microbes and their biotic and abiotic environments. Within these functional networks, certain species and strains are considered to be keystone and have been postulated to play crucial roles in the functional ecology of the environment (Barberán *et al.*, 2012; Bulgarelli *et al.*, 2012; van der Heijden and Hartmann, 2016). Since the present results showed that the inoculation of *M. pudica* with *P. phymatum* significantly affected the microbial communities associated with root surfaces, we attempted to clarify whether keystone species were associated with these changes. Therefore, we analyzed and compared the bacterial network and keystone taxa associated with inoculated and uninoculated roots using co-occurrence network analyses (Berry and Widder, 2014; Banerjee *et al.*, 2018). The network of co-occurring OTUs was comprised of 924 nodes and 1,330 edges. The keystone taxa within each network of positive co-occurrences were comprised of OTUs belonging to two different genera: *Streptomyces* and *Phycicoccus* (Fig. 7). Members of *Streptomyces* significantly increased from 0.079% in the uninoculated treatment to 0.113% in the inoculated treatment (Tukey’s HSD, *P*<0.05), while those of *Phycicoccus* significantly decreased from 0.101 to 0.061% (Fig. 7). The increase observed in *Streptomyces* may be of ecological significance for nodulation by *P. phymatum* because these bacteria synthesize and secrete diverse secondary metabolites that include many antibiotics (van Kulen and Dyson, 2014). In the plant rhizosphere, *Streptomyces* spp. have been shown to produce antifungal and plant growth-promoting metabolites and have potential as inoculants for enhancing agriculture production (Schrey and Tarkka, 2008; Rey and Dumas, 2017). Further studies are needed to clarify the direct/indirect effects of *Streptomyces* on legume nodulation in non-sterile soils.

### *P. phymatum* predominantly occupies *M. pudica* nodules

**Fig. 8.**
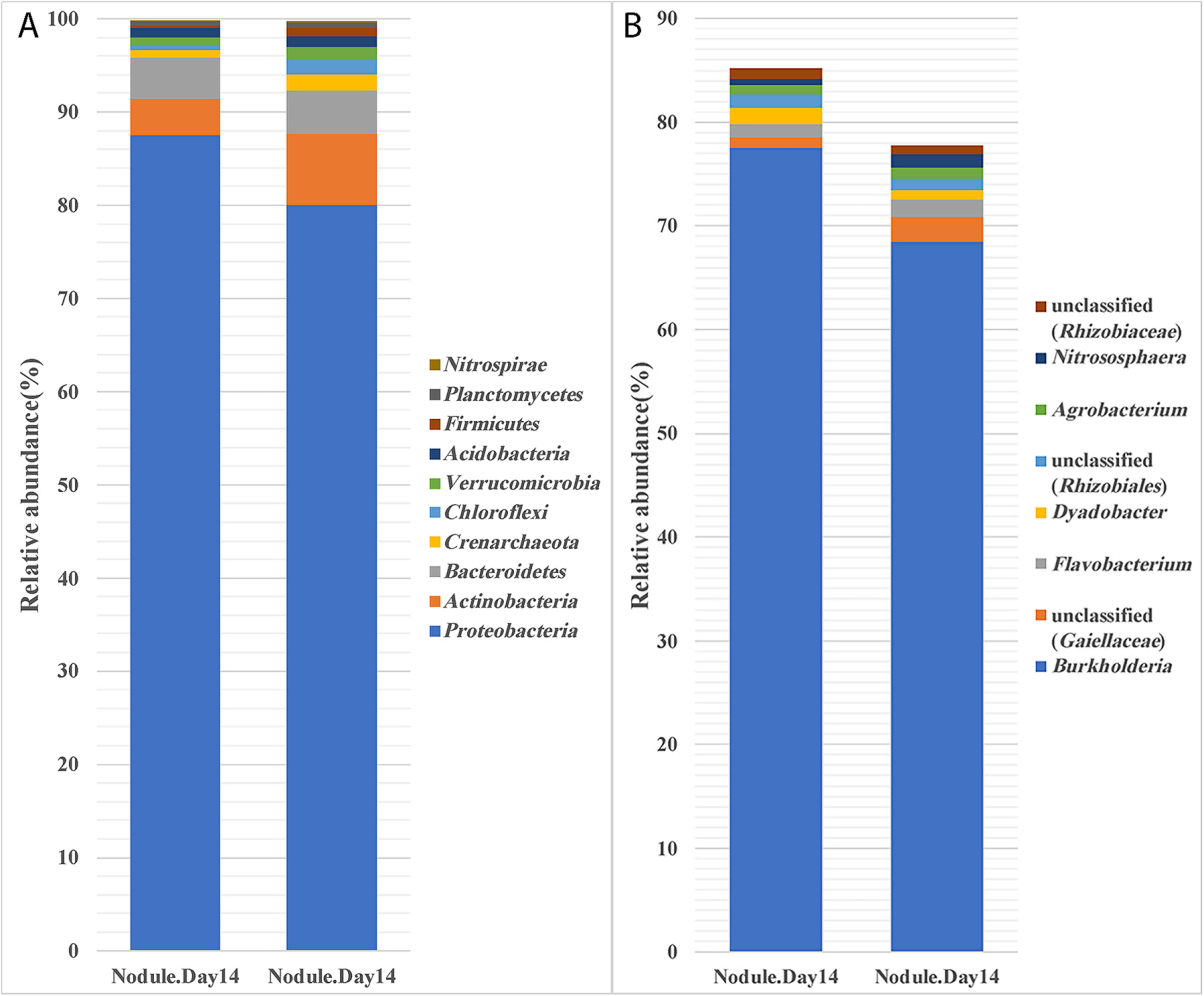
Relative abundance of *Paraburkholderia phymatum* in nodules of *Mimosa pudica*. (A) At the phylum level, ~80% of OTUs were *Proteobacteria*. (A) At the genus level, ~75% OTUs were (para) *Burkholderia*.

Previous studies showed that bacteria unrelated to legume-nodulating rhizobia may be isolated from surface-sterilized nodules, indicating that other bacteria also possess the ability to infect legume nodules (Deng *et al.*, 2011; Aserse *et al.*, 2013; Martinez-Hidalgo and Hirsch, 2017). Although these bacteria do not nodulate under axenic conditions, they may be important for symbiosis in soil environments. Studies conducted to date have relied on the isolation of culturable bacteria from nodules. Since the majority of bacteria are not cultured in the laboratory, the extent of the non-rhizobial occupants of nodules in soils remains unclear. In the present study, we analyzed the 16S rRNA diversity of *M. pudica* nodules that formed following an inoculation with *P. phymatum*. While the results obtained demonstrated that the majority (~75%) of nodule-occupant OTUs were from *Burkholderia*, which was expected, we also detected OTUs from *Agrobacterium*, *Rhizobiales*, *Nitrososphaera*, and *Flavobacterium* in the homogenates of surface-sterilized nodules (Fig. 8B). Since no nodules were detected on *M. pudica* in the absence of the *P. phymatum* inoculation, these bacteria do not have the ability to nodulate *M. pudica*. Therefore, they may have entered *M. pudica* nodules via a co-infection mechanism. This phenomenon is now well recognized (Muresu *et al.*, 2008; Martinez-Hidalgo and Hirsch, 2017). However, further studies are needed to examine the role played by these co-infecting microbes.

## Conclusion

We examined the effects of an inoculation of *P. phymatum* on changes in the microbial community in the rhizoplane, roots, and nodules in natural agricultural soil. An analysis using the 16S rRNA gene showed that *P. phymatum* did not significantly change the community of the rhizospheric soil. In contrast, the population of *P. phymatum* significantly increased in root samples. A network analysis identified *Streptomyces* spp. as keystone taxa that may be involved in nodulation by *P. phymatum*.

## Citation

Welmillage, S. U., Zhang, Q., Sreevidya, V. S., Sadowsky, M. J., and Gyaneshwar, P. (2021) Inoculation of *Mimosa Pudica* with *Paraburkholderia phymatum* Results in Changes to the Rhizoplane Microbial Community Structure. *Microbes Environ ***36**: ME20153.

https://doi.org/10.1264/jsme2.ME20153

## Supplementary Material

Supplementary Material
