# Supplementary material for: Inoculation of Mimosa Pudica with Paraburkholderia phymatum Results in Changes to the Rhizoplane Microbial Community Structure
Source: Microbes Environ. 2021 Mar 12;36(1):ME20153. doi: 10.1264/jsme2.ME20153 (PMC7966945; doi:10.1264/jsme2.ME20153)

Supplementary Data

Fig. S1. Experimental design to study effects of a *Paraburkholderia phymatum* inoculation on the bacterial community in the rhizosphere of *Mimosa pudica*. Seedlings of *M. pudica* were grown in pots containing a 1:1 mixture of vermiculite and soil. Rhizosphere, root, and nodule samples were collected from inoculated and uninoculated samples and used in a bacterial community analysis.

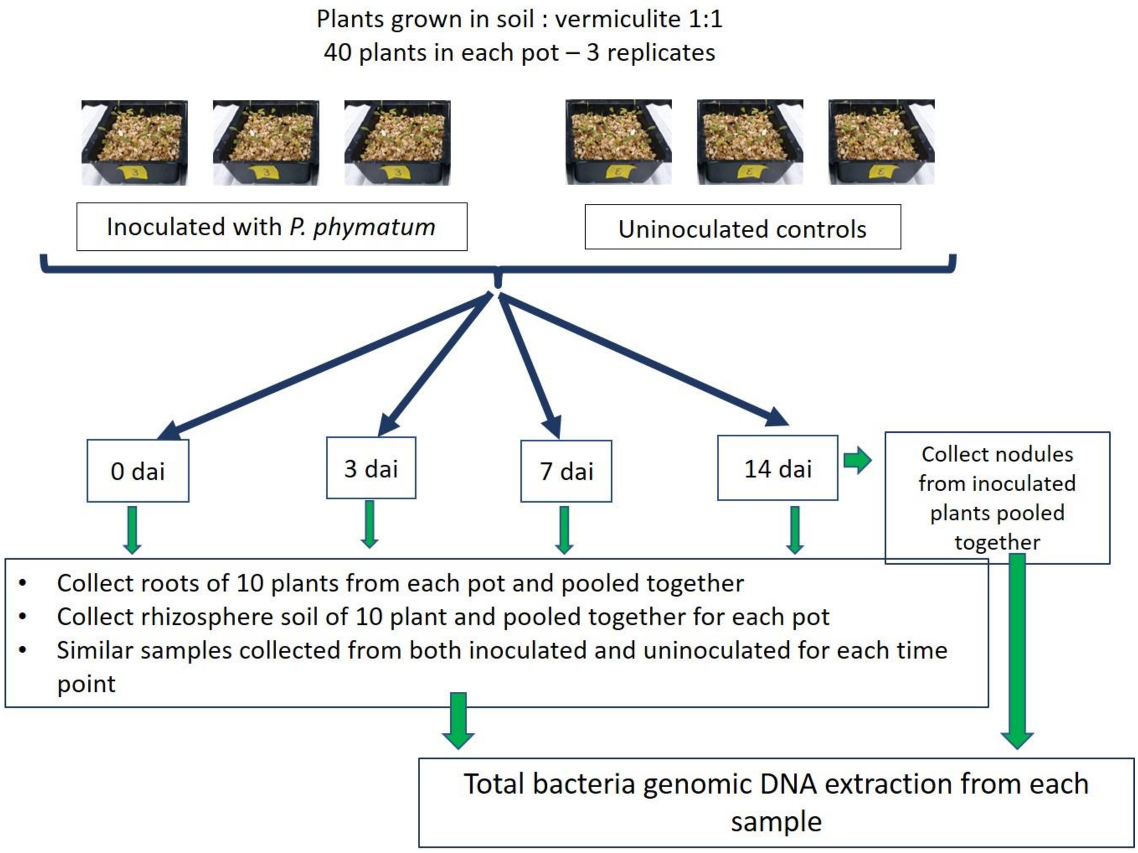

Supplement: Supplementary file 1 — Supplementary Material [file 36_20153_s1.pdf]
